# Crystal Structure of the PIM2 Kinase in Complex with an Organoruthenium Inhibitor

**DOI:** 10.1371/journal.pone.0007112

**Published:** 2009-10-20

**Authors:** Alex N. Bullock, Santina Russo, Ann Amos, Nicholas Pagano, Howard Bregman, Judit É. Debreczeni, Wen Hwa Lee, Frank von Delft, Eric Meggers, Stefan Knapp

**Affiliations:** 1 University of Oxford, Structural Genomics Consortium, Oxford, United Kingdom; 2 Swiss Light Source, Paul Scherrer Institut, Villigen PSI, Switzerland; 3 Fachbereich Chemie, Philipps-Universität Marburg, Hans-Meerwein-Strasse, Marburg, Germany; 4 Department of Chemistry, University of Pennsylvania, Philadelphia, Pennsylvania, United States of America; University of Cambridge, United Kingdom

## Abstract

**Background:**

The serine/threonine kinase PIM2 is highly expressed in human leukemia and lymphomas and has been shown to positively regulate survival and proliferation of tumor cells. Its diverse ATP site makes PIM2 a promising target for the development of anticancer agents. To date our knowledge of catalytic domain structures of the PIM kinase family is limited to PIM1 which has been extensively studied and which shares about 50% sequence identity with PIM2.

**Principal Findings:**

Here we determined the crystal structure of PIM2 in complex with an organoruthenium complex (inhibition in sub-nanomolar level). Due to its extraordinary shape complementarity this stable organometallic compound is a highly potent inhibitor of PIM kinases.

**Significance:**

The structure of PIM2 revealed several differences to PIM1 which may be explored further to generate isoform selective inhibitors. It has also demonstrated how an organometallic inhibitor can be adapted to the binding site of protein kinases to generate highly potent inhibitors.

**Enhanced version:**

**This article can also be viewed as an enhanced version in which the text of the article is integrated with interactive 3D representations and animated transitions. Please note that a web plugin is required to access this enhanced functionality. Instructions for the installation and use of the web plugin are available in Text S1.**

## Introduction

The PIM2 kinase belongs to a family of three serine/threonine kinases (PIM1-3) first identified as preferential proviral insertion sites in Moloney Murine Leukemia Virus (MoMuLV) induced T-cell lymphomas [1], [2]. In humans PIM2 has been implicated in the transformation of both T and B lymphocytes and is highly expressed in human leukemia and lymphomas [3]. Importantly, expression of the *pim2* transgene predisposes mice to T-cell lymphomas and is highly cooperative with the Eμ-*myc* transgene in the development of pre-B cell leukaemia [4]. Located on the X chromosome the pim2 gene is highly induced by growth factors and cytokines through STAT5 activation. Indeed its downstream activation by oncogenes including JAK2, v-ABL and FLT3-ITD appears essential for their ability to drive tumorigenesis [5]–[7]. For example, cells transformed by FLT3 or BCR/ABL mutations that confer resistance to small-molecule inhibitors remain sensitive to PIM2 knockout by RNAi [8].

PIM kinases confer a growth advantage through a variety of mechanisms. They promote growth factor-independent proliferation by phosphorylation of cell cycle factors such as p21^Cip1/Waf1^ [9], cdc25A [10] and eIF4e-BP1 [11]. They protect cells from apoptosis by phosphorylation of the pro-apoptotic protein BAD [12]. The PIM1 kinase has also been shown to phosphorylate an ABC transporter [13] promoting drug efflux and to co-activate MYC-target genes by phosphorylation of histone H3 serine 10 [14]. PIM2 also confers resistance to rapamycin indicating a parallel signaling pathway from the PI3K/Akt/TOR cascade [11], [15].

The PIM2 kinase has therefore emerged as a key drug target to restore apoptosis in drug resistant human cancers [16]. To date structural information for the PIM kinase family is restricted to PIM1 for which the majority of inhibitor development has also been directed [17]–[20]. Interestingly, PIM kinases have an altered hinge region which does not allow the formation of two hydrogen bonds to ATP typically present in protein kinase ATP complexes. The presence of a proline residue in the PIM hinge sequence (ERPXPX) removes the typical +3 hydrogen bond donor of the hinge backbone resulting in considerably high Km values for ATP. Inhibitors often mimic these hydrogen bonds leading to considerable cross-reactivity with other kinases that all share this active site feature. Thus, the considerably different active site of PIM kinases provides potential for the design of PIM-specific inhibitors. Subsequently, a series of imidazo[1,2-b]pyridazine inhibitors was identified with anti-leukemic activity that bound PIM1 in an ATP competitive but non-ATP mimetic manner [21]. Surprisingly, PIM2 was markedly less susceptible to inhibition than PIM1.

**Figure 1:**
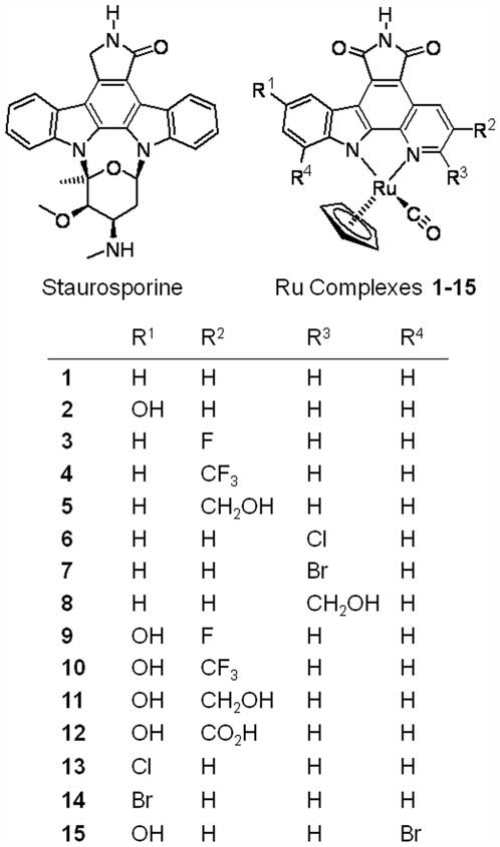
Staurosporine as an inspiration for the design of organoruthenium complexes 1–15. Shown is the (R)-isomer.

We have developed a series of metal complexes inspired by the staurosporine scaffold [22] that enable us to expand the available small molecule chemical space and identify new inhibitors of PIM2. In the designed organoruthenium complexes the coordinate bonds are proven to be kinetically inert and are therefore likely to be stable in vivo thus avoiding metal-related toxicity. Here, we describe the crystal structure of human PIM2 bound to one of these inhibitors, the (*R*)-enantiomer of compound **1** (Fig. 1). In combination with our inhibition data, the structure and specificity profiles highlight the view of the metal centre as a “hypervalent carbon” and further extend structural opportunities for inhibitor design.

## Results

### Overview of the PIM2 structure

**Figure 2:**
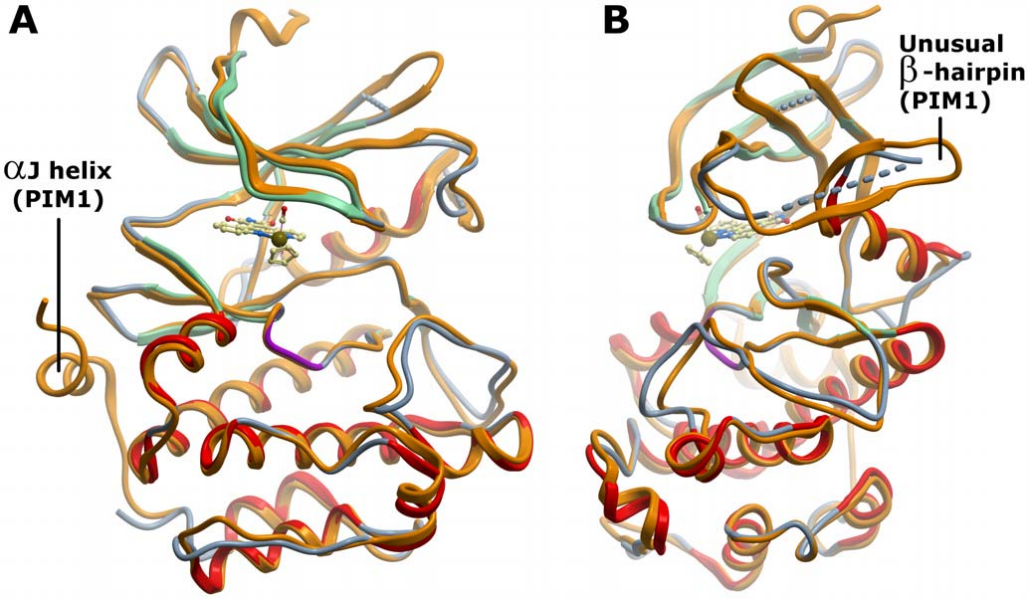
Overall structure of PIM2 and comparison with PIM1. Overlay of the two proteins (shown in ribbon representation) reveals the strong conservation of the kinase fold. A. PIM1 (2bzh, coloured orange) contains the C-terminal αJ helix that is absent in PIM2 (coloured green for β-strand and red for α-helix). B. The view is rotated by 90° to highlight the unusual β-hairpin in the kinase N-lobe which is partially disordered in the PIM2 structure.

The PIM2 protein crystallized in spacegroup H3 with two protein molecules per asymmetric unit and was refined to 2.8 Å resolution (see Table 1 for data collection and refinement statistics). Overall, PIM2 shows the typical bilobal kinase architecture with a constitutively active closed conformation. The main chain of both molecules is identical with the exception of two flexible regions in the N-terminal lobe. At the N-terminus residues Gly22 to Glu31 which are disordered in molecule A form a short helix in molecule B while the loop preceding the αC helix is disordered in molecule B (Asn66 to Val78), but partially ordered in molecule A. In addition, the tip of the adjacent β4-β5-loop is not defined in either molecule (Fig. 2).

**Table 1:** Crystallographic data and refinement statistics.

**Data collection**	pdb-code: 2IWI
Space group	H3
Cell dimensions [Å]	a = 154.770, b = 154.770, c = 78.600
Resolution [Å]	2.8
Unique reflections	16017
Completeness**	99.69 (95.2)
R_merge_	4.9%
I/σ**	21.0 (2.1)
**Refinement**	
R_work_ (R_free_ *) (%)	24.4/28.9
Rmsd bond length [Å]	0.012
Rmsd bond angle [^o^]	1.265
Average B-factor (Å^2^)	35.2
Protein atoms	3687
Other	95
**Ramachandran**	
allowed [%]	91.1
generously allowed [%]	8.9
dissallowed [%]	0

* using randomly selected 5% of data.

** values in last shell shown in brackets.

### Structural differences between PIM1 and PIM2

**Figure 3:**
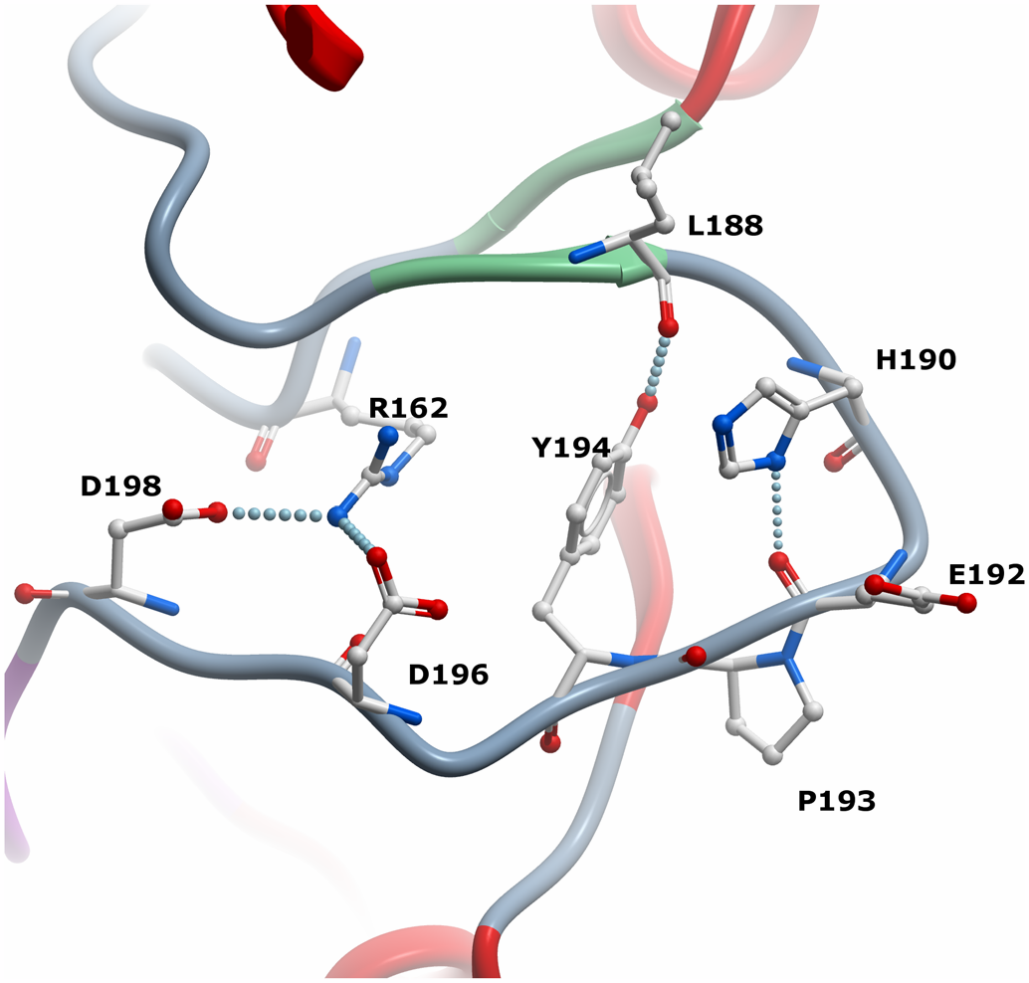
Activation loop structure in PIM2. Residues stabilizing a constitutively active loop conformation via hydrogen bonding are shown in stick representation.

Human PIM2 shares 55% sequence identity with PIM1. Overall the PIM2 structure is similar to the closely related PIM1 structure (PDB 2BZH, [23]) with the main chain atoms superimposing with an r.m.s.d. of 0.9 Å. The PIM1 hinge-region sequence ERPEPV is conservatively replaced by ERPLPA and both kinases lack the typical +3 hydrogen bond donor. The activation loops also show similar active conformations with a conserved aspartate (PIM2 Asp196) mimicking the phosphorylated Thr288 in active Aurora-A (Fig. 3). In PIM2 both the Asp196 and Asp198 side chains form salt bridge interactions with Arg162 from the catalytic HRD motif, although this side chain was disordered in molecule A. The activation segment is also stabilized by hydrogen bonds formed between Tyr194 and the main chain of Leu188 (as well as His190 in molecule A) and between Asp192 and the side chain of His190. However, the salt bridge between the conserved αC Glu83 and the active site lysine (Lys61) is not observed due to disorder of the lysine side chain. Substrate binding residues identified from the structure of PIM1 in complex with a consensus peptide (RRRHPSG) [24] are also strictly conserved in PIM2 consistent with their overlapping substrate specificity.

The most significant difference between PIM1 and PIM2 is the absence of the C-terminal αJ helix in PIM2 (Fig. 2A). The last 23 residues of PIM2 share little sequence identity with PIM1 and are disordered. This PIM2 region contains 6 proline residues and is not predicted to form helical structure. In PIM1 αJ packs below the β8-β9-loop and the absence of this interaction might increase flexibility in PIM2 within the N-terminal kinase lobe and contribute to the disordered regions of the PIM2 structure. One of these regions, the loop preceding helix αC, forms an unusual β-hairpin in PIM1 (Fig. 2B). Some dozen residues are present in this insertion in all three PIM kinases and the partial structure present in PIM2 molecule A suggests a similar architecture. Additionally, the loop following αC in both PIM2 and PIM3 contains a two-residue insertion relative to PIM1 which changes the loop conformation. In PIM2 this loop is potentially destabilized by the sequence G-A-G-G-G which may further increase flexibility in the N-terminal lobe.

### Inhibitor synthesis and design

The organoruthenium complexes mimic the highly potent inhibitor staurosporine with a distinctive globular structure more similar to the shape of the kinase ATP pocket than many planar kinase inhibitors. The indolocarbazole alkaloid scaffold is replaced with a simple metal complex that retains the main features of the indolocarbazole aglycon in a metal-chelating pyridocarbazole ligand while the carbohydrate is replaced by a ruthenium fragment. Utilizing new chemical space, this scaffold has shown remarkable specificity for the PIM1 kinases [23] and glycogen synthase kinase 3 (GSK-3) [25].

### Binding mode of compound 1

**Figure 4:**
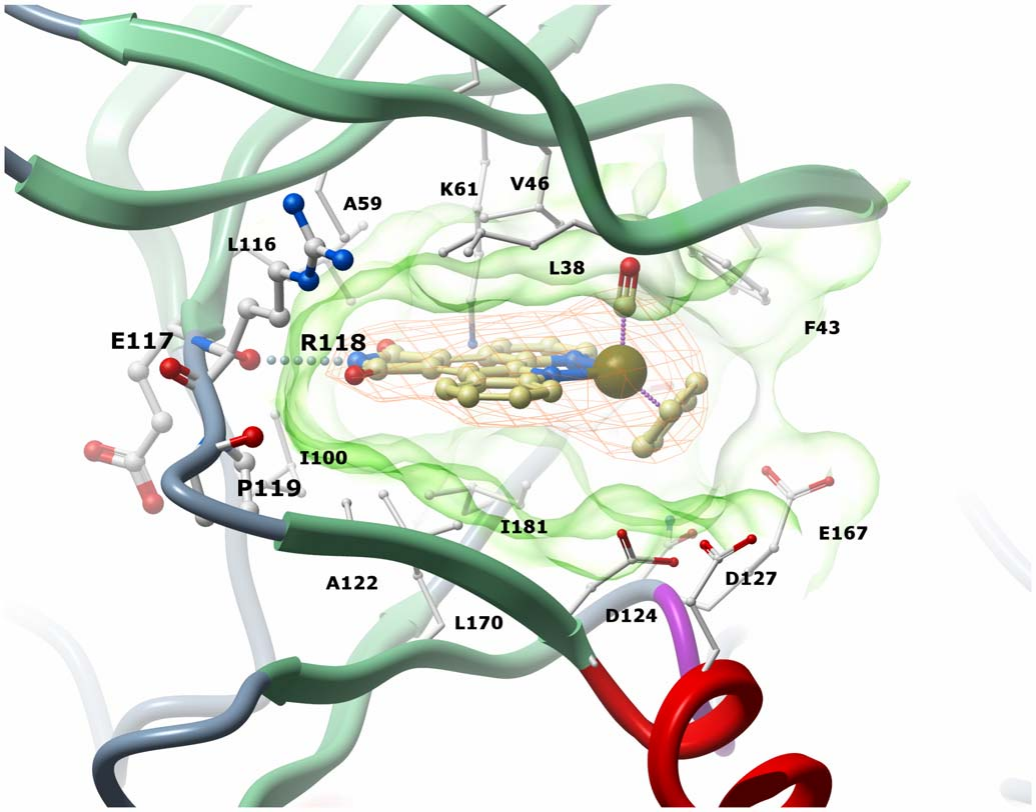
Inhibitor interactions in the ATP pocket. The surface of the ATP pocket is shown in transparent green. The electron density (2FoFc, contoured at 1σ level) for inhibitor 1 is shown in orange.

The structure of PIM2 in complex with the (*R*)-enantiomer of compound 1 [23], [26] shows the perfect fit of the inhibitor to the ATP pocket (Fig. 4). As designed the metal centre does not form any direct interactions with the kinase domain but plays a structural role organizing the organic ligands in the three-dimensional space. Overall, the binding mode is conserved compared to the structure of PIM1 in complex with the same inhibitor (2BZH) [23]. The hinge region proline (Pro119) restricts PIM2 to the formation of only one hydrogen bond with ATP and ATP mimetic inhibitors. As expected, the maleimide of compound 1 establishes one hydrogen bond between the imide NH and the backbone carbonyl oxygen of Glu117. An additional water-mediated hydrogen bond in the equivalent PIM1-inhibitor complex between the maleimide carbonyl and the backbone amide of Glu186 from the DFG motif (PIM2 Glu182) is not observed in PIM2, but could reflect the lower resolution of the structure.

A key feature of both the PIM1 and PIM2 complexes is the close contact of the CO ligand with the glycine rich loop (the distance in PIM2 between the Gly39 carbon alpha and the CO group is just 3.1 Å) in which Gly39 together with Val46 and Phe43 in PIM2 form a small hydrophobic pocket for the CO ligand. The tight binding of the inhibitor is further explained by the close hydrophobic packing on the N-terminal lobe face from Leu38, Phe43, Val46, Ala59 and Leu116 and on the opposite face from Ile100, Ala122, Leu170, Ile181. All of these residues are conserved in PIM1 with the exception of Ala122 which is replaced by Val126 in PIM1. This results in a small increase in the inhibitor packing distance which is 3.5 Å from the PIM1 valine side chain and 4.0 Å from the PIM2 alanine. The adjacent hinge substitution from PIM1 Glu124 to PIM2 Leu120 could also introduce a subtle change in the dynamics of the PIM hinge region, but does not change the hinge structure. This region contains a two-residue insertion relative to most kinases and consequently makes no interaction with the inhibitor.

### Structure-activity relationship (SAR) of Ru-based inhibitors

**Figure 5:**
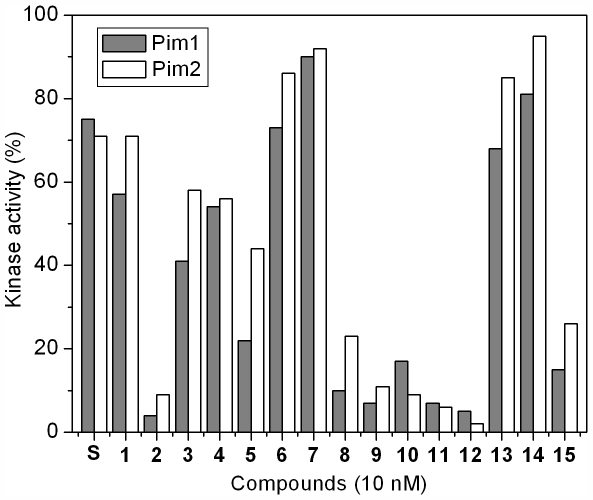
Screening of racemic ruthenium complexes 1–15 against PIM1 and PIM2 at a concentration of 10 nM. Staurosporine (S) was used as a reference.

The scaffold of compound **1** was further explored with an additional 14 derivatives [27] (Fig. 1) and SAR was performed against PIM1 and PIM2 using a radiolabeled in vitro phosphorylation assay (Fig. 5). In the presence of either staurosporine or the crystallized inhibitor **1** PIM2 retained ∼70% activity at an inhibitor concentration of 10 nM. The SAR suggests that the addition of potential hydrogen bonding groups at the R1 and R2 positions dramatically increases potency against both kinases. Similar substitution of the R3 position was less effective and halogen substitution was even more disruptive. The majority of compounds were slightly more potent against PIM1 than PIM2. However, the most potent inhibitor for PIM2, compound **12**, which gave almost complete inhibition at a concentration of 10 nM, was marginally less effective against PIM1.

## Discussion

The proto-oncogene PIM2 is a key mediator of hematopoietic cell growth and apoptotic resistance and complements transformation by c-MYC and mutant tyrosine kinases including BCR/ABL and FLT3-ITD. Importantly, PIM2 inactivation can restore apoptosis to otherwise drug-resistant cancers and is therefore an attractive therapy to supplement current drug regimes such as Gleevec™. The structure of PIM2 reveals a constitutively active conformation consistent with the view that PIM2 activity is regulated principally at the transcriptional level [11]. Consequently, the oncogenic potential of PIM2 is greatly increased on overexpression.

Overall, the structure is highly similar to PIM1, particularly in the ATP pocket which is nearly completely conserved in comparison to the overall sequence identity of 55%. The generally reduced susceptibility of PIM2 to previously characterized PIM1 inhibitors such as LY331′531 [28] might instead result from a change in protein dynamics as suggested here by several disordered loops in the N-terminal kinase lobe. The main structural distinction between the two kinases is the absence of the αJ helix in PIM2 which removes a significant stabilizing interaction close to the interface between the N and C-terminal lobes as well as differences in the kinase hinge and P loop residues.

Based on the initial staurosporine scaffold the organoruthenium complexes have provided marked specificity for the GSK3 and PIM kinases by the introduction of the metal centre coordinated by a cyclopentadienyl ring and a CO ligand [23], [25], [26]. The structures of PIM1 and now PIM2 bound to **1** show a remarkable fit between the inhibitor and the ATP pocket that explains the inhibitor's potency. Our SAR analysis highlights the promise for further scaffold optimization with both kinases having particular preference for a hydroxyl substituent at the R1 position (compound **2**) [27], [29]. The structure of PIM1 in complex with compound 2 showed similar positions for the maleimide group, the cyclopentadienyl ring and the CO ligand, but a 180° flip in the pyridocarbazole moiety that enables two water-mediated hydrogen bonds to form through the R1 hydroxyl with Glu89 [23].

This flexibility indicates further opportunity for inhibitor derivatisation and indeed PIM2 was inhibited most strongly by compound 12 containing an additional carboxyl group at the R2 position. Interestingly, the inhibitor LY331′531 also bound PIM1 in two conformations (PDB 2J2I [28]) and the imperfect fit may partially explain its ineffectiveness against PIM2. The primary LY331′531 conformation makes close contact with PIM1 Val126 (3.3 Å) and the subtle change to Ala122 in PIM2 may be sufficient to destabilize this binding mode. The PIM kinases contain a two-residue insertion in the hinge preceding this position and the smaller PIM2 side chain may allow greater exploitation of this available space.

Mouse knockouts lacking all three PIM genes remain viable and fertile but show reduced body size with no hematopoietic response to growth factors [30]. The PIM2 structure and inhibitor data presented here provide further direction to develop well-tolerated drug molecules that stop growth factor independence, limit drug resistance and induce tumour apoptosis.

## Materials and Methods

### Protein expression and purification

Full length human PIM2 (34 kD isoform, gi 42821112) was subcloned by ligation-independent cloning into a pET-derived expression vector, pLIC, and expression performed in BL21(DE3) with 0.15 mM isopropyl 1-thio-β-D-galactopyranoside induction for 4 h at 18°C. Cells were lysed using a high pressure homogenizer and cleared by centrifugation, and the lysate was purified by nickel-sepharose chromatography. The eluted PIM2 protein was treated with λ-phosphatase together with tobacco etch virus (TEV) protease overnight to remove phosphorylation and the hexahistidine tag, respectively. The protein was further purified on a Mono Q column and by size exclusion chromatography. The eluted protein was homogeneous and non-phosphorylated as shown by ESI-MS. PIM2 protein was stored at 4°C in elution buffer (50 mM HEPES, pH 7.5, 250 mM NaCl) with 10 mM DTT or frozen in liquid nitrogen and stored at −80°C. Typical crystals had dimensions of 15×5×5 µm^3^.

### Crystallization and Structure Determination

PIM2 was concentrated to 11 mg/ml in the presence of compound **1** which was added to an initial concentration of 0.6 mM (from a 10 mM stock solution in DMSO). Crystals were grown at 4°C in 1.5 µl sitting drops mixing 0.3 µl PIM2 with 1.2 µl mother liquor (90 mM HEPES pH 7.5, 1.44 M Na/KPO_4_) and cryo-protected in mother liquor containing 30% glycerol.

PIM2 diffraction data were collected on a flash-cooled crystal (100 K) at the Swiss Light Source beamline SLS X10SA. Images were indexed and integrated using MOSFLM, and scaled using SCALA implemented in the CCP4 suite of programs. The structure was solved by molecular replacement using the program Phaser with the coordinates of PIM1 in complex with BIM1 (Protein Data Bank (PDB) code 1XWS). REFMAC5 was used for refinement with iterative rounds of rigid-body refinement and restrained refinement with TLS, against maximum likelihood targets, interspersed with manual rebuilding of the model using Xfit/XtalView.

Coordinates for the PIM2-inhibitor complex have been deposited in the Protein Data Bank (PDB code 2IWI).

### Measurement of Protein Kinase Inhibition

The synthesis of all compounds has been reported recently [26], [27]. PIM kinases (human) and substrate were purchased from Upstate Biotechnology USA. 10 nM concentrations of staurosporine or compounds 1–15 were incubated at room temperature in 20 mM MOPS, 30 mM MgCl_2_, 0.8 µg/µL BSA, 5% DMSO (resulting from the inhibitor stock solution), pH 7.0, in the presence of substrate (50 µM S6 kinase/Rsk2 substrate peptide 2) and kinase (3.3 nM PIM1, 1.5 nM PIM2). After 15 minutes, the reaction was initiated by adding ATP to a final concentration of 100 mM, including approximately 0.2 µCi/µL [γ-^32^P]ATP. Reactions were performed in a total volume of 25 µL. After 45 minutes, the reaction was terminated by spotting 17.5 µL on a circular P81 phosphocellulose paper (2.1 cm diameter, Whatman), followed by washing four times (5 minutes each wash) with 0.75% phosphoric acid and once with acetone. The dried P81 papers were transferred to a scintillation vial, and 5 mL of scintillation cocktail was added, and the counts per minute (CPM) were determined with a Beckmann 6000 scintillation counter. Each compound was measured in duplicate. Percent activity was calculated by dividing the averaged CPM for each compound by the control sample, corrected by the background.

### Note

For further details about this kinase strutcure, please refer to SGC Material and Methods entry for PIM2.

## Supporting Information

Datapack S1 Standalone iSee datapack - contains the enhanced version of this article for use offline. This file can be opened using free software available for download at http://www.molsoft.com/icm_browser.html.
(ICB)

Text S1 Instructions for installation and use of the required web plugin (to access the online enhanced version of this article).
(PDF)
